# TRPM7 in CHBP-induced renoprotection upon ischemia reperfusion-related injury

**DOI:** 10.1038/s41598-018-22852-2

**Published:** 2018-04-03

**Authors:** Aifen Liu, Jing Wu, Cheng Yang, Yuanyuan Wu, Yufang Zhang, Fengbo Zhao, Hui Wang, Li Yuan, Lirui Song, Tongyu Zhu, Yaping Fan, Bin Yang

**Affiliations:** 1 Renal Group, Basic Medical Research Centre, Medical College of Nantong University, Nantong, Jiangsu 226001 China; 2 Department of Nephrology, Affiliated Hospital of Nantong University, Nantong, Jiangsu 226001 China; 3 Department of Urology, Zhongshan Hospital, Fudan University; Shanghai Key Laboratory of Organ Transplantation, Shanghai, 200032 China; 4 Department of Pathology, Medical College of Nantong University, Nantong, Jiangsu 226001 China; 5 Shanghai Institute of Materia Medica, Chinese Academy of Sciences, Shanghai, 201203 China; 6 University of Chinese Academy of Sciences, Beijing, 100049 China; 7 Department of Infection, Immunity and Inflammation, University of Leicester, Leicester General Hospital, University Hospital of Leicester, Leicester, LE1 9HN United Kingdom

## Abstract

Transient receptor potential melastatin 7 (TRPM7) is a membrane ion channel and kinase. TRPM7 was abundantly expressed in the kidney, and up-regulated by ischemia reperfusion (IR) injury. Our previous studies showed that cyclic helix B peptide (CHBP) improved renal IR-related injury, but its underlying mechanism is not well defined. IR-related injury was established in renal tubular epithelial cells (TCMK-1 and HK-2) via 12 to 24-h hypoxia (H) followed by 2-24 h reoxygenation (R), and in mouse kidneys subjected to 30-min ischemia and 12-h to 7-day reperfusion. TRPM7-like current in TCMK-1 cells, TRPM7 mRNA and protein in the *in vitro* and *in vivo* models were increased, but reversed by CHBP. TRPM7 was also positively associated with LDH, HMGB1, caspase-3, Bax/Bcl-2, inflammation, apoptosis, tubulointerstitial damage and renal function respectively. Furthermore, silencing TRPM7 improved injury parameters, renal histology and function in the both models. Specific TRPM7 agonist, bradykinin, exaggerated HR induced injury in TCMK-1 cells, and partially blocked the renoprotection of CHBP as well. In conclusion, TRPM7 is involved not only in IR-related injury, but also CHBP-induced renoprotection, which are through its ion channel and subsequent affects inflammation and apoptosis. Therefore, TRPM7 could be a potential biomarker for IR-induced acute kidney injury.

## Introduction

Ischemia reperfusion (IR) injury is one of the major causes of acute kidney injury (AKI), increases the risk of delayed allograft dysfunction and rejection in transplant kidneys, and may also lead to chronic kidney diseases (CKD)^1–4^. It is imperative to find new preventive and therapeutic strategies for IR-related AKI to reduce its morbidity,  mortality and progression to CKD. Our previous studies revealed that caspase-3 activation, inflammation and apoptosis occurred in renal IR-related injuries^5,6^, while Bax/Bcl-2 balance associated with caspase-3 modulated inflammation and apoptosis^7^. Furthermore, we also proved that the inhibition of caspase-3, as well as high mobility group box-1 protein (HMGB1), another inflammation and apoptosis associated molecule marker, protected kidneys in a variety of IR-related injury models^8–10^. However, the mechanism and prevention/treatment of renal IR-related injury are still need to be further investigated.

Transient receptor potential melastatin 7 (TRPM7), as an ion channel and kinase, has been attracted a great attention from many scientists since it was reported in 2001 by Runnels^11^. TRPM7 is ubiquitously expressed in most organs^12,13^ and has multiple functions. The notable feature of TRPM7 is the permeation of Ca^2+^, Mg^2+^ and trace metals, suggesting its possible role to depolarize the cells and increase intracellular calcium^11,12^. As a kinase, TRPM7 phosphorylates some substrate molecules such as annexin I, myosin II and m-calpain^14^. In the recent years, TRPM7 has been found involved in IR neuronal cell death^15,16^ and myocardial injuries^17^. Moreover, Meng *et al*. reported that the expression of TRPM7 was up-regulated in early stage of renal IR-related injury^18^ and the suppression of renal TRPM7 may alleviate kidney injury in renal transplantation^19^.

Erythropoietin (EPO) is mainly secreted by foetal livers and adult kidneys^20^. Besides its role in hematopoietic system, recent interests have been fueled by its tissue protection in various organs^21–23^. Our studies also demonstrated the renoprotection of EPO in porcine tubular proximal cells and rat renal IR models^20,24^. However, the renoprotective effects need high dose of EPO, which might cause unsatisfied side effects such as hypertension and tumorigenesis. Helix B surface peptide (HBSP) is a linear peptide derived from EPO without erythropoietic function^25^. Our previous studies proved the renoprotective effects of HBSP in kidney IR models^26,27^. However, due to instability of linear construction, the half-life of HBSP is very short, which restricts its clinical translation. We recently synthesized a novel thioether cyclic helix B peptide (CHBP), which has significantly improved metabolic stability and potent renoprotective effects^28–32^. The role of TRPM7 in CHBP-induced renoprotection upon IR-related injury is unknown.

In the present study, we first demonstrated that the expression of TRPM7 was upregulated in the hypoxia and reoxygenation (HR) treated mouse TCMK-1 cells and IR mouse kidneys; then revealed that TRPM7 expression and TRPM7-like current were inhibited by CHBP. There was a positive correlation between TRPM7 level, cellular damage and tissue injury in both *in vitro* and *in vivo* models. TRPM7 siRNA decreased TRPM7 expression and suppressed TRPM7-like current, and further reduced inflammation and apoptosis in HR TCMK-1 cells and mouse IR kidneys. In addition, specific TRPM7 agonist, bradykinin^33,34^, raised TRPM7 and then increased inflammation and apoptosis, while TRPM7 activation by bradykinin partially blocked CHBP-induced renoprotection. Further, TRPM7 expression was also positively associated with inflammation and apoptosis injuries in HR TCMK-1 cells and mouse IR kidneys.

## Results

### TRPM7 expression increased in HR tubular cells and IR kidneys

**Figure 1:**
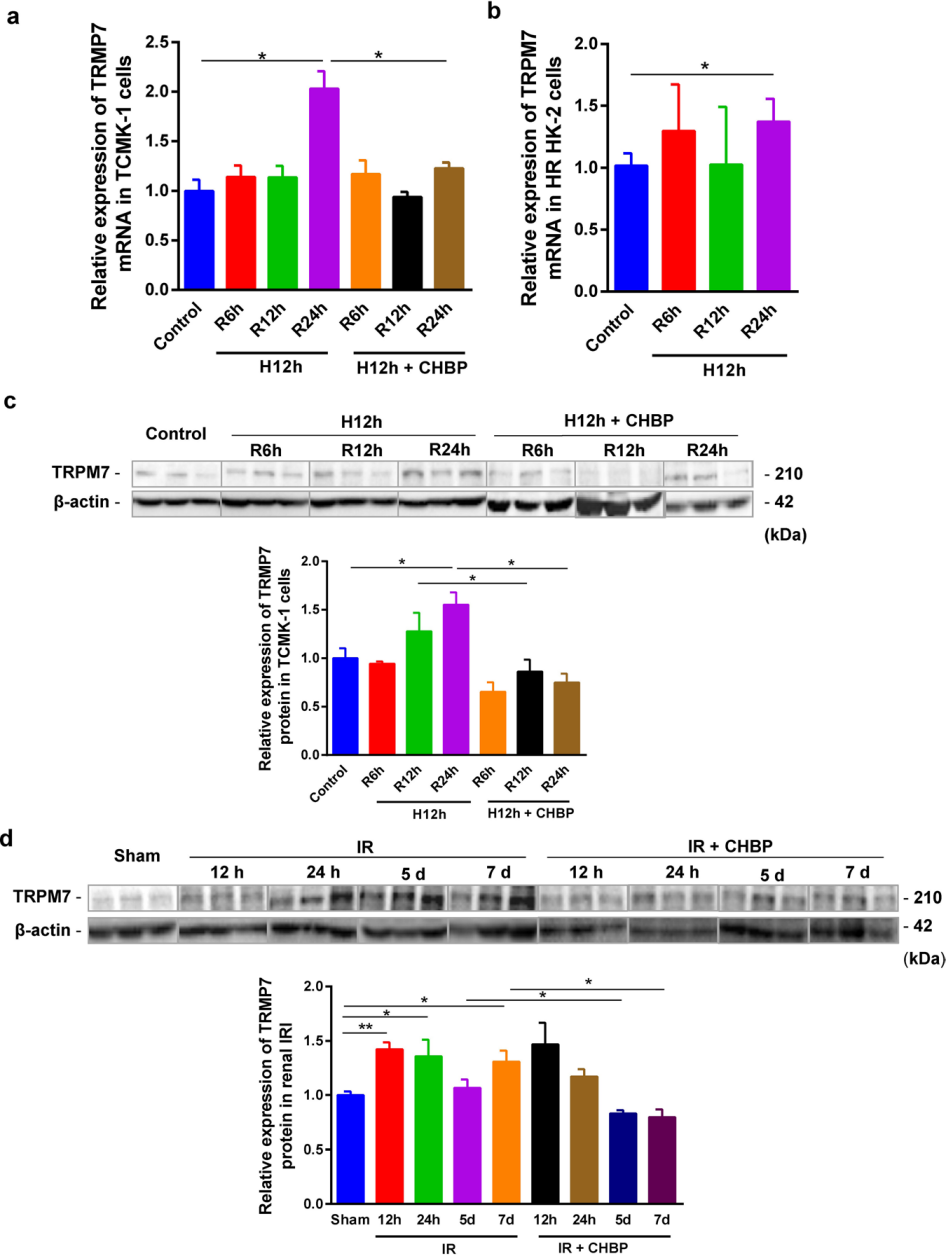
The inhibited role of CHBP on increased TRPM7 at mRNA and protein levels in HR cells and IR kidneys. TRPM7 mRNA level was increased by 24-h reperfusion in both TCMK-1 (**a**) and HK-2 cells (**b**) subjected to 12-h HR, While TRPM7 protein level was also increased in TCMK-1 cells subjected to 24-h reoxygenation in 12-h H TCMK-1 cells (**c**), and in the mouse kidneys followed 12 h, 24 h, and 7 days reperfusion (**d**). In addition, the expression of TRPM7 mRNA (A) and protein (**c**) was reduced in the 12-h, 24-h HR TCMK-1 cells by 30 nM CHBP at the onset of hypoxia. The TRPM7 protein was significantly reduced by 8 nmol/Kg CHBP intraperitoneally injected in the 5-d, 7-d IR mouse kidneys (**d**). The volume density was quantitatively analyzed using 42 kDa β-actin as the loading control (n = 6).

The expression of TRPM7 mRNA in TCMK-1 cells, detected by real-time RT-PCR, was increased 2.0 folds at 24 h HR, but not at 6 or 12 h (Fig. 1a), compared with the non-hypoxic control. A similar change trend of TRPM7 mRNA was revealed in HK-2 cells (Fig. 1b), with slightly less than 2-fold increase at 24-h HR compared with the non-hypoxia group, but there was no statistical difference at 6 and 12 h.

The level of TRPM7 protein in TCMK-1 cells was measured by Western blot. Semi-quantitative analysis revealed that the expression of TRPM7 protein was significantly increased by 30% and 33% in 12-h and 24-h HR, compared with the non-hypoxic control, with a statistical significance only at 24 h (Fig. 1c). In addition, the concurrent increase of TRPM7 protein was revealed in a mouse renal IR model from 12 h to 7 d. Compared with the sham group, the statistical significance was demonstrated at 12 h, 24 h and 7 d (Fig. 1d).

### CHBP inhibited TRPM7 expression in HR TCMK-1 cells and IR kidneys

To evaluate the effect of CHBP on TPRM7 expression in mouse HR TCMK-1 cells and IR kidneys, we detected the mRNA and protein expression of TRPM7 subjected to the treatment of 30 nM CHBP. In the TCMK-1 cells, increased TRPM7 mRNA at 24 h was reduced by 40% (Fig. 1a), while increased TRPM7 protein was significantly reduced by 32% and 52% at 12 h and 24 h in the CHBP treated group, respectively (Fig. 1c). In mouse kidneys treated with CHBP, TRPM7 protein was still significantly higher at 12 h and 24 h compared with the sham group, whereas it was significantly reduced at later time points 5 d and 7 d (Fig. 1d).

### TRPM7 correlated with injury in both *in vitro* and *in vivo* models

**Figure 2:**
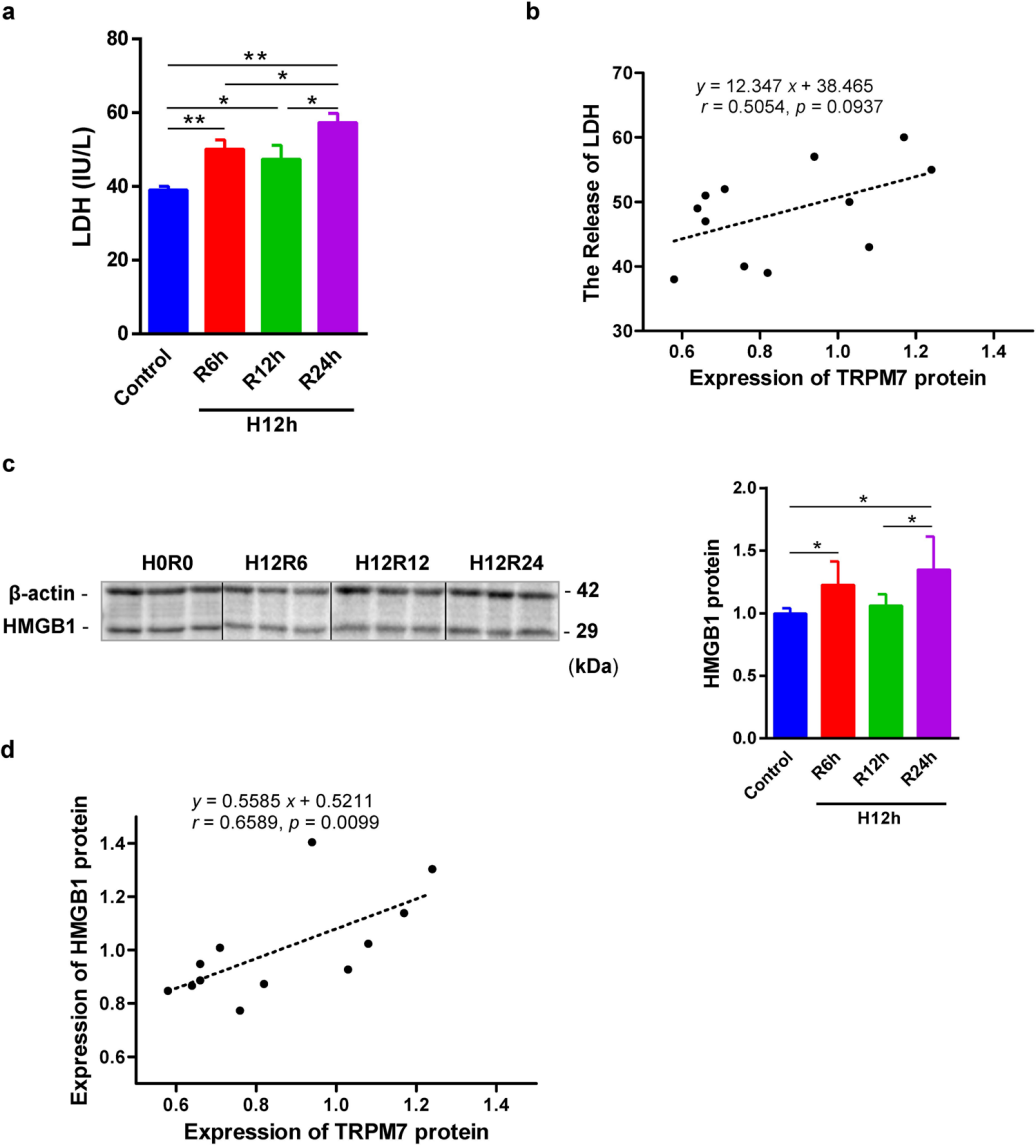
The positive correlation between TRPM7 protein, supernatant LDH and cellular HMGB1 in HR TCMK-1 cells. Cytotoxicity was assessed by LDH release in TCMK-1 cells exposed to 12-h H and followed by 6-h, 12-h, and 24-h R (**a**). The positive correlation between TRPM7 expression and LDH in TCMK-1 cells (**b**). The expression of HMGB1 was also detected by western blotting in HR TCMK-1 cells (**c**). The positive correlation between TRPM7 and HMGB1 protein was revealed in TCMK-1 cells (n = 6, (**e**)).

LDH and HMGB1 are biomarkers of injury, which releases from damaged tissues or cells. The release of LDH was significantly raised in the supernatant of HR TCMK-1 cells at 6 and 24 h with a peak at the later (Fig. 2c). The expression of TRPM7 protein and the level of supernatant LDH in TCMK-1 cells were marginally correlated (*P* = 0.093, Fig. 2b). The expression of HMGB1 was increased in HR TCMK-1 cells at 6 and 24-h, and peaked at 24 h (Fig. 2c). In addition, the expression of HMGB1 and TRPM7 protein in TCMK-1 cells were strongly related to each other (*P* = 0.0099, Fig. 2d).

**Figure 3:**
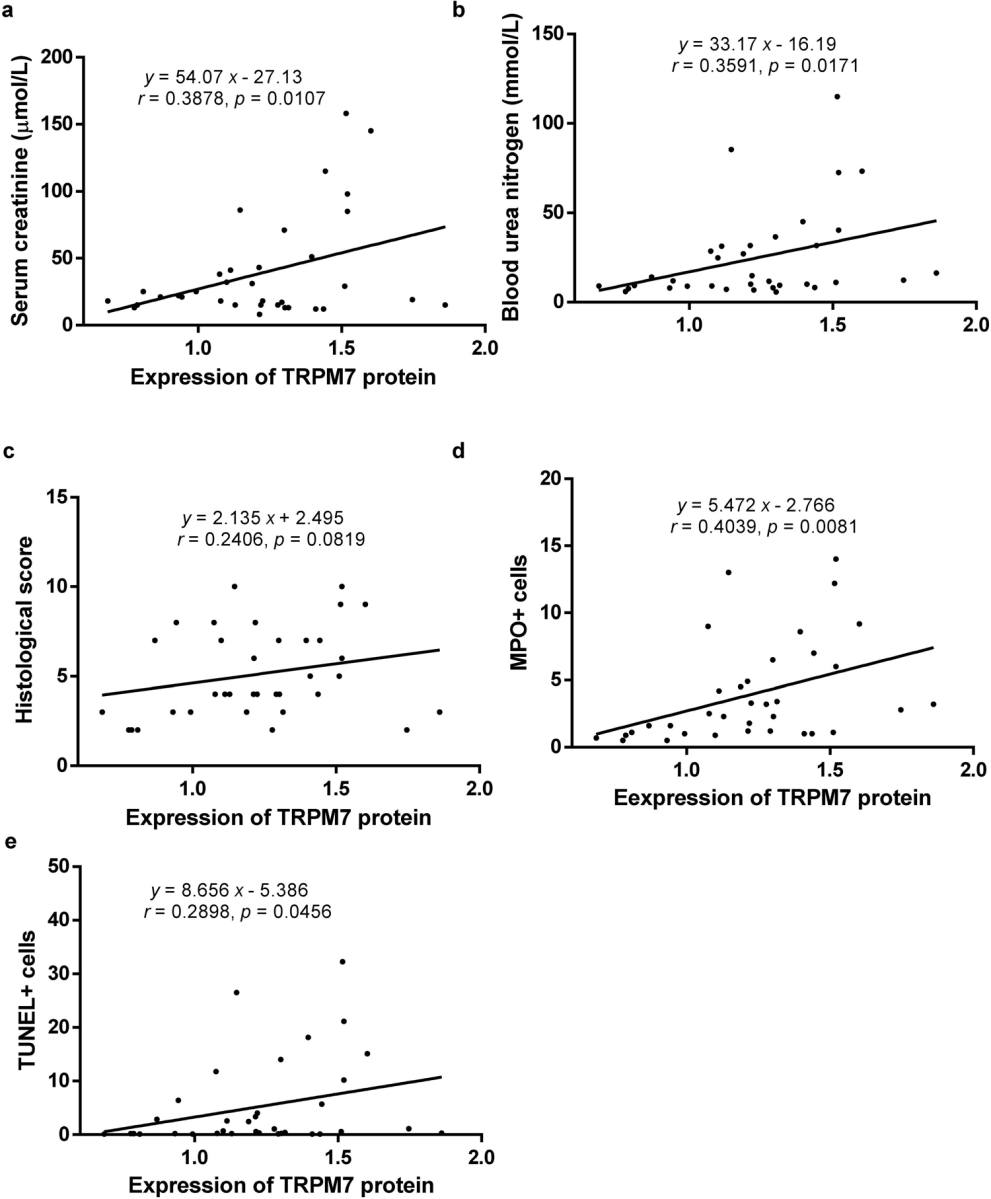
The positive correlation between TRPM7 protein and parameters of renal IR injury. TRPM7 expression was positively related to serum creatinine (**a**), blood urea nitrogen (**b**), inflammation (**d**) and apoptosis (**e**) in mouse IR injury kidneys, only marginally associated with tubulointerstitial damage score (**c**).

Here, we further showed that TRPM7 protein expression was positively associated with serum creatinine, blood urea nitrogen, inflammation and apoptosis respectively, only marginally associated with the score of tubulointerstitial damage (Fig. 3).

### CHBP inhibited TRPM7-like current in TCMK-1 cells

**Figure 4:**
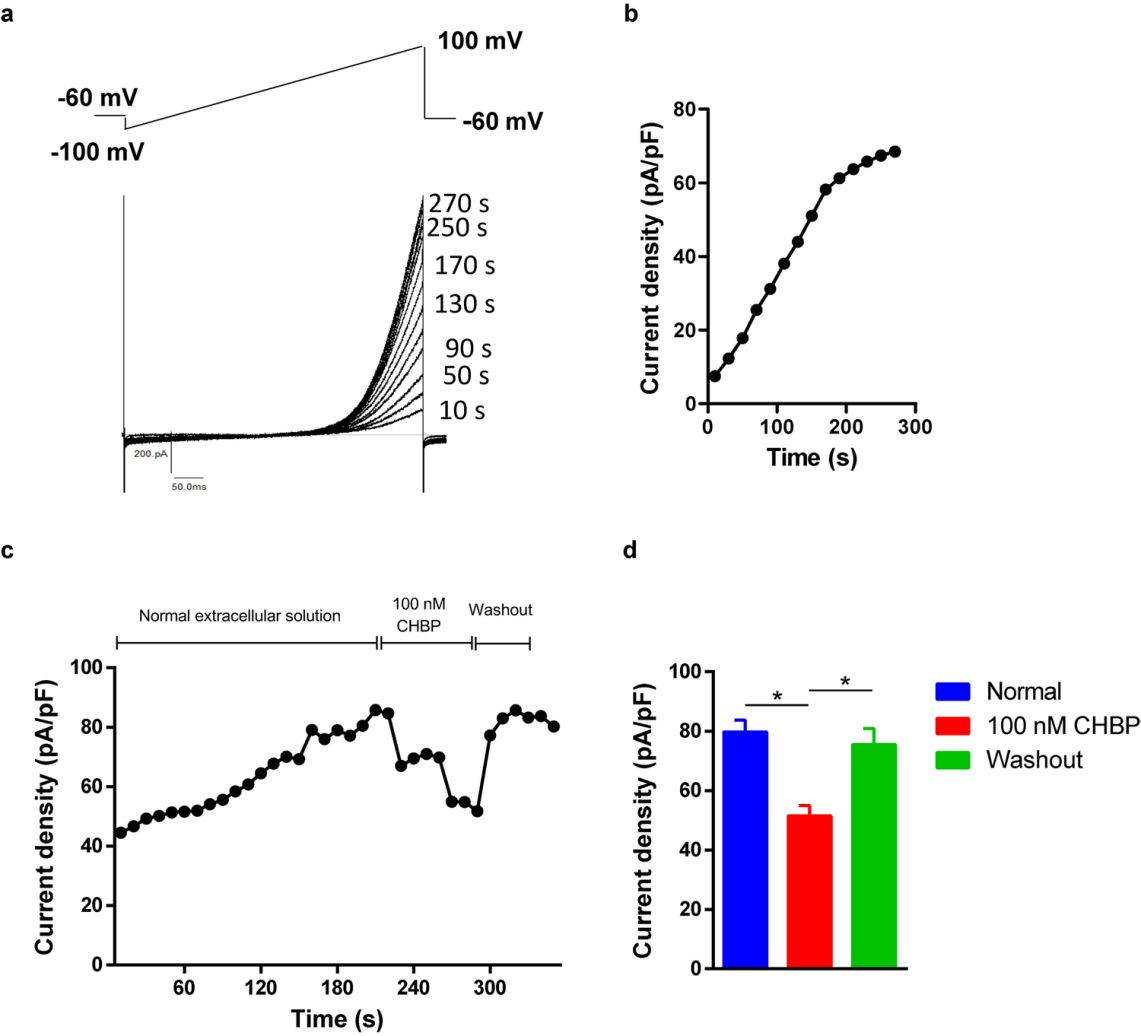
CHBP inhibited TRPM7-like currents in TCMK-1 cells. The voltage ramp protocol invoking TRPM7 currents and the representative TRPM7 currents recorded in TCMK-1 cells (**a**). Time-dependent running up of TRPM7-like currents after break-in when dialyzed with Mg^2+^ free internal solution by whole cell patch clamp (**b**). Blockade of TRPM7-like current (at +100 mV) by CHBP (100 nmol/l) (**c**). The periods of exposure to the normal extracellular solutions and CHBP were indicated by horizontal bars. Outward TRPM7-like currents were inhibited by CHBP, but reversibly changed by CHBP washout. (n = 6, (**d**)).

TRPM7-like currents were generated in TCMK-1 cells using a previously validated protocol^35,36^. Under whole-cell configuration, typical outward rectified TRPM7-like currents could be recorded in TCMK-1 cells (Fig. 4a). After membrane rupture under the whole-cell configuration, both inward and outward TRPM7-like current was time-dependently increased using pipettes filled with internal Mg^2+^-free solution (Fig. 4a,b). In order to clarify the effects of CHBP on TRPM7-like currents, the stabilized TRPM7 current was recorded after membrane rupture at 220 s and then TRPM7 currents was observed in TCMK-1 cells perfused with normal extracellular solution, with or without 100 ng/ml CHBP. TRPM7 current was induced progressively changes as the time increased with CHBP treatment (Fig. 4c). Compared with normal group, the outward TRPM7-like currents was maximum reduced by 46% with CHBP treatment (Fig. 4d).

### TRPM7 siRNA downregulated its mRNA and TRPM7-like current in TCMK-1 cells

**Figure 5:**
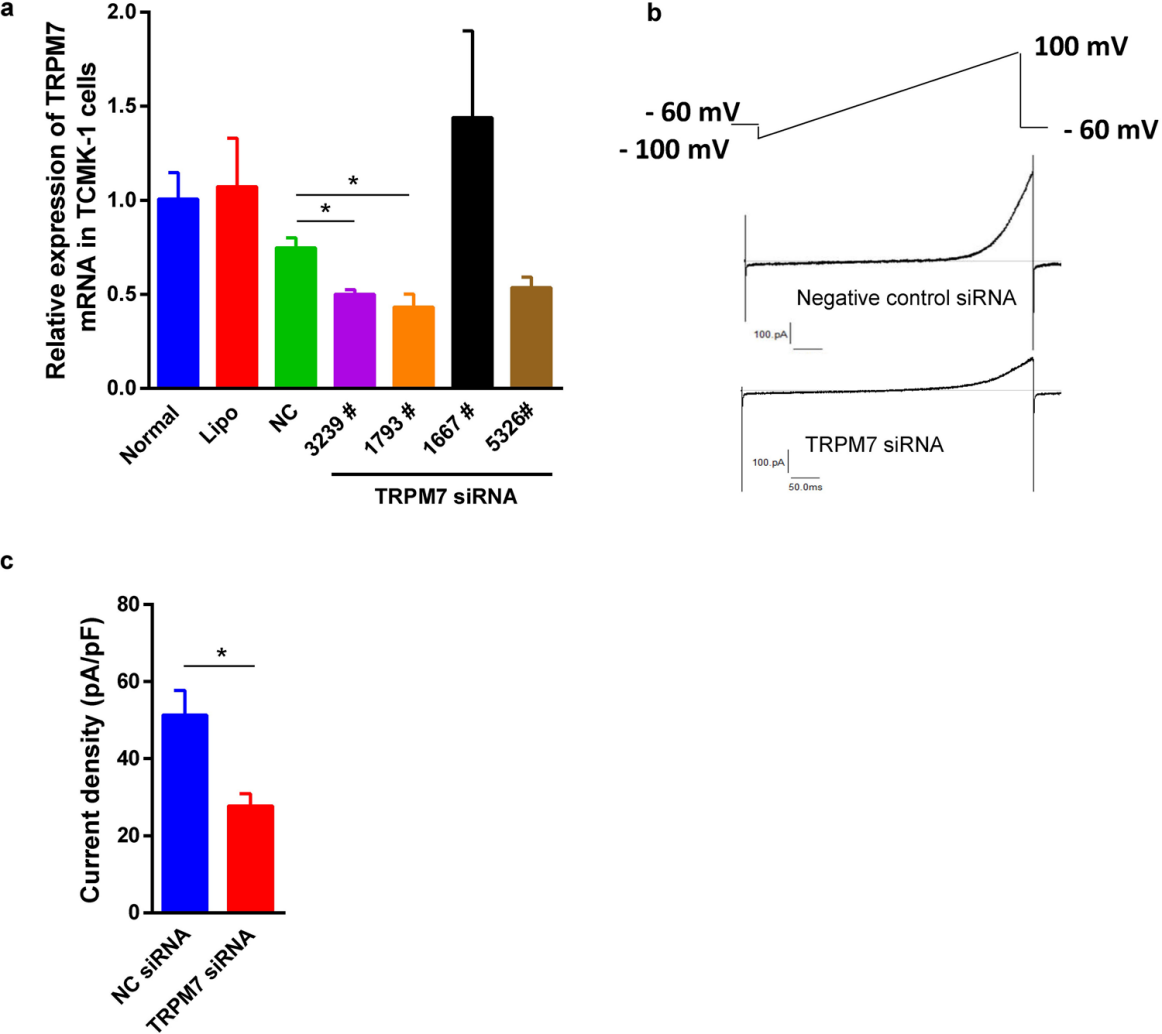
TRPM7 siRNA downregulated its mRNA expression and TRPM7-like current in TCMK-1 cells. Compared to the cells treated by the NC siRNA, the expression of TRPM7 mRNA was reduced by TRPM7 siRNA sequence 3239 and 1793 in TCMK-1 cells (*P* < 0.05 (**a**). However, TRPM7 siRNA 5326 and 1667 did not statistically decrease TRPM7 mRNA expression. The representative whole cell TRPM7-like currents invoked by the voltage ramp protocol were recorded in TCMK-1 cells transfected with NC siRNA and TRPM7 siRNA 1793 (**b**). The outward TRPM7-like currents were inhibited by the sequence 1793 of TRPM7 siRNA (**c**).

The expression of TRPM7 mRNA in the TCMK-1 cells transfected with TRPM7 siRNAs with sequence number 3239, 1793 and 5326 was reduced by 33%, 42% and 28% respectively compared to the cells treated by the negative control (NC) siRNA (Fig. 5a). However, TRPM7 siRNA sequences 5326 and 1667 did not significantly reduce TRPM7 expression. TRPM7 siRNA 1793, therefore, was selected for further functional study. In order to explore the impact of TRPM7 siRNA on its downstream biological functions, TRPM7 siRNA 1793 sequence at 40 nM for 48 h caused a noticeable reduction of TRPM7 current in comparison to cells treated with NC siRNA (Fig. 5b,c).

### Silencing TRPM7 in TCMK-1 cells reduced inflammation and apoptosis

**Figure 6:**
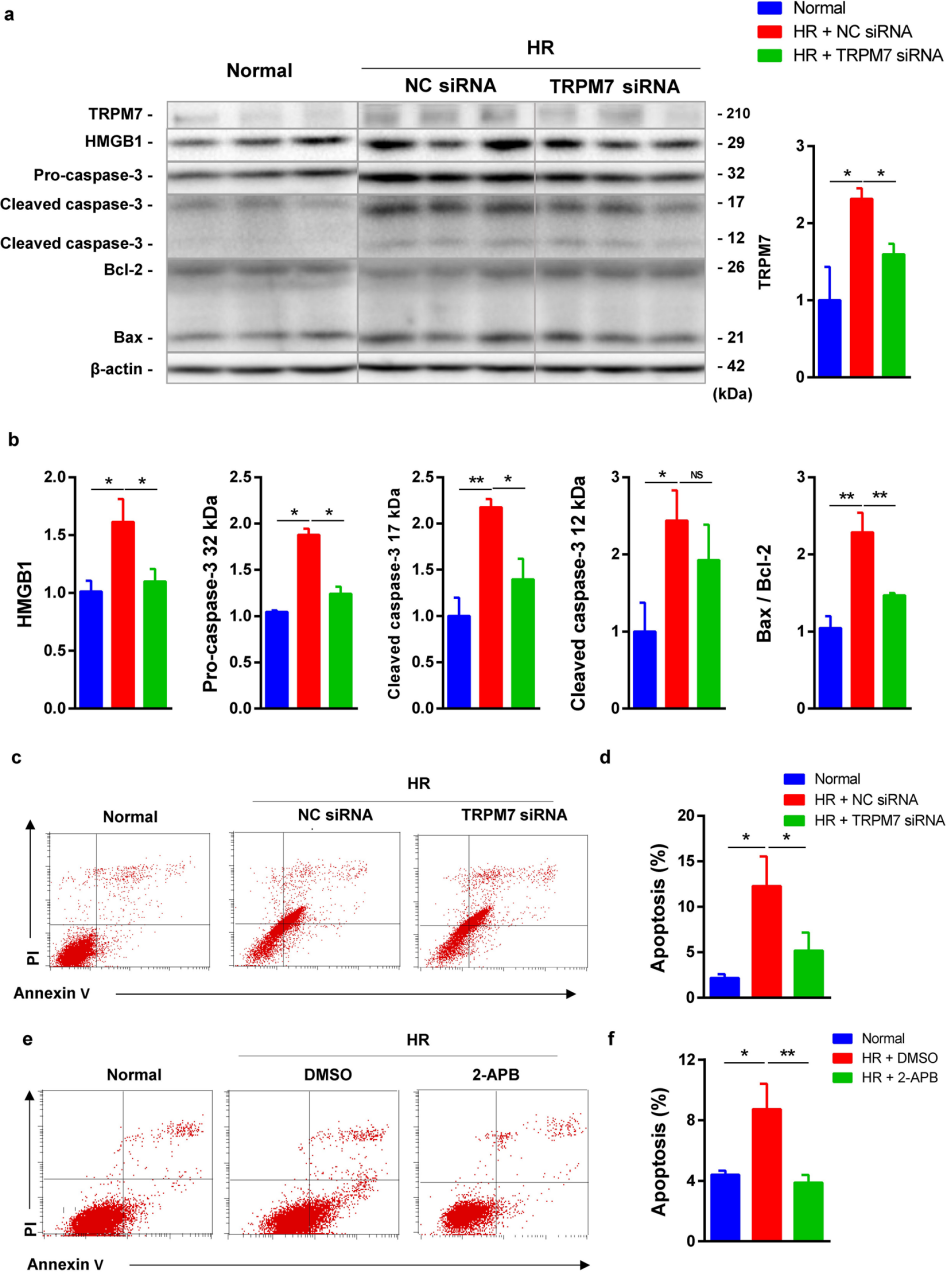
TRMP7 siRNA and inhibitor reduced inflammation and apoptosis inTCMK-1 cells. The protein expression of TRPM7, HMGB1, 32, 17 and 12 kDa caspase-3, Bax and Bcl-2 in the HR TCMK-1 cells was detected by western blotting (**a**). TRPM7 siRNA significantly reversed increased TRPM7, HMGB1, 32 and 17 kDa caspase-3 and Bax/Bcl-2 ratio, but not 12 kDa caspase-3 upon HR (**b**). Apoptotic cells were labelled by Annexin V/PI and quantitatively assessed by flow cytometer (**c**,**e**). The histograms showed that the increase of early apoptotic cells was inhibited by TRPM7 siRNA and its inhibitor 2-APB (n = 3, (**d**,**f**)).

The effect of TRPM7 siRNA on inflammation and apoptosis was further investigated. TCMK-1 cells were subjected to 24-h hypoxia and followed by 2-h reoxygenation treated with/without TRPM7 siRNA 1793 or TRPM7 inhibitor 2-aminoethoxydiphenyl borate (2-APB). Compared to the NC siRNA, TRPM7 siRNA reduced TRPM7 protein, decreased inflammation and apoptosis-related HMGB1, 32 kDa and 17 kDa caspase-3 protein, as well as Bax/Bcl-2 ratio (Fig. 6a,b). Flow cytometry assay using Annexin V/PI labelling revealed a significant decrease of 49.4% and 65% in the portion of Annexin V(+)/PI(−) TCMK-1 cells treated by TRPM7 siRNA and 2-APB respectively compared to the control group (Fig. 6c–f).

### Silencing TRMP7 ameliorated renal IR injury

**Figure 7:**
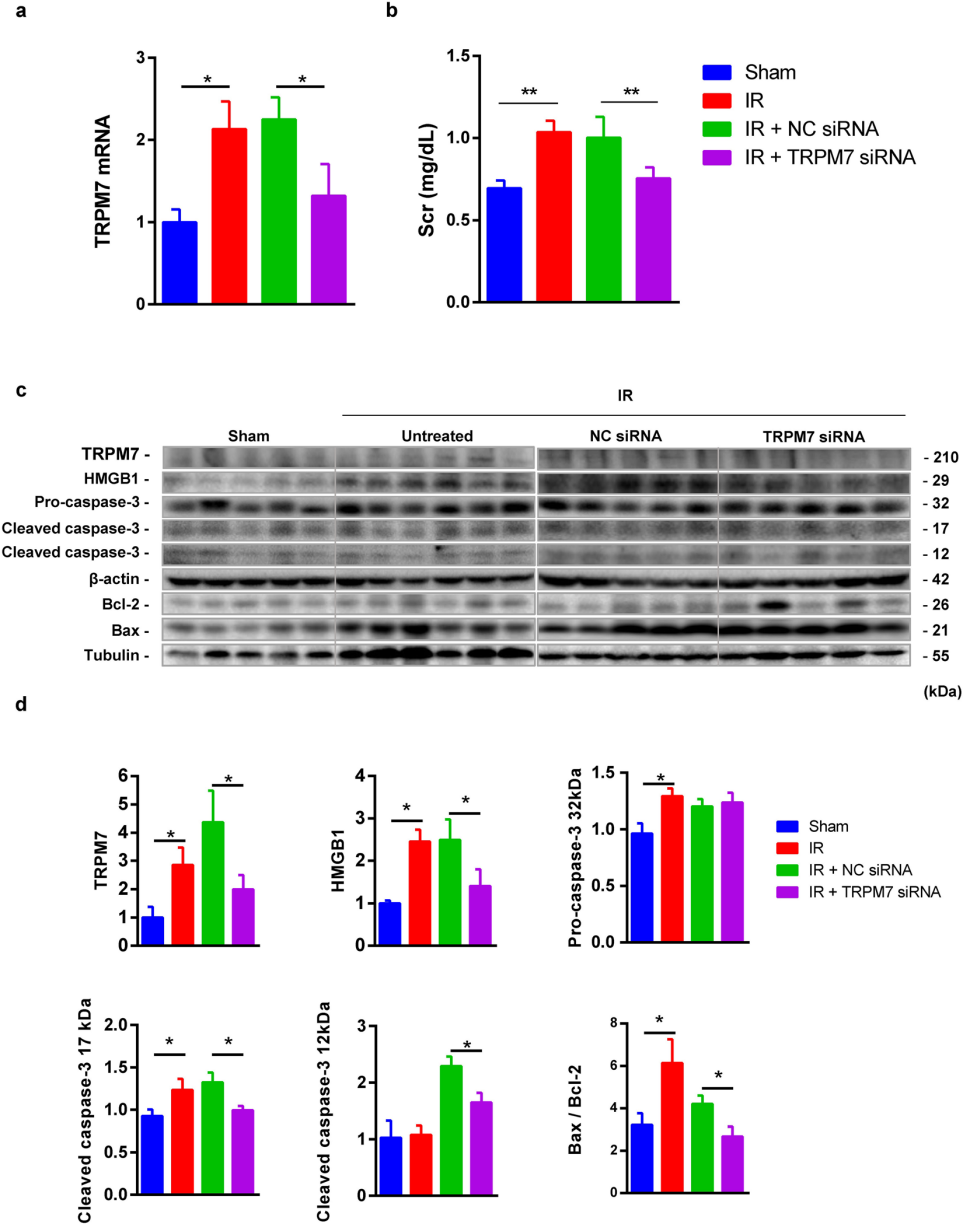
TRPM7 siRNA inhibited inflammation and apoptosis in mouse IR kidneys. TRPM7 siRNA decreased the expression of TRPM7 mRNA (**a**) and the level of serum creatinine (**b**). TRPM7, HMGB1, 32, 17 and 12 kDa caspase-3, Bax and Bcl-2 were detected by western blotting (**c**). In IR kidneys, TRPM7 siRNA significantly down-regulated TRPM7, HMGB1, 17 and 12 kDa caspase-3 and Bax/Bcl-2 ratio, but not 32 kDa caspase-3 (**d**).

The effect of down-regulated TRPM7 by siRNA on renal IR injury was further evaluated. Compared to the sham group, TRPM7 mRNA and protein, serum creatinine, HMGB1, 32 kDa and 17 kDa caspase-3, as well as Bax/Bcl-2 ratio were all increased in the IR group. However, compared to the NC siRNA group, TRPM7 siRNA inhibited the increased TRPM7 mRNA and protein, serum creatinine, HMGB1, 17 and 12 kDa caspase-3 and Bax/Bcl-2 ratio (Fig. 7).

### Activating TRPM7 increased inflammation and apoptosis in TCMK-1 cells

**Figure 8:**
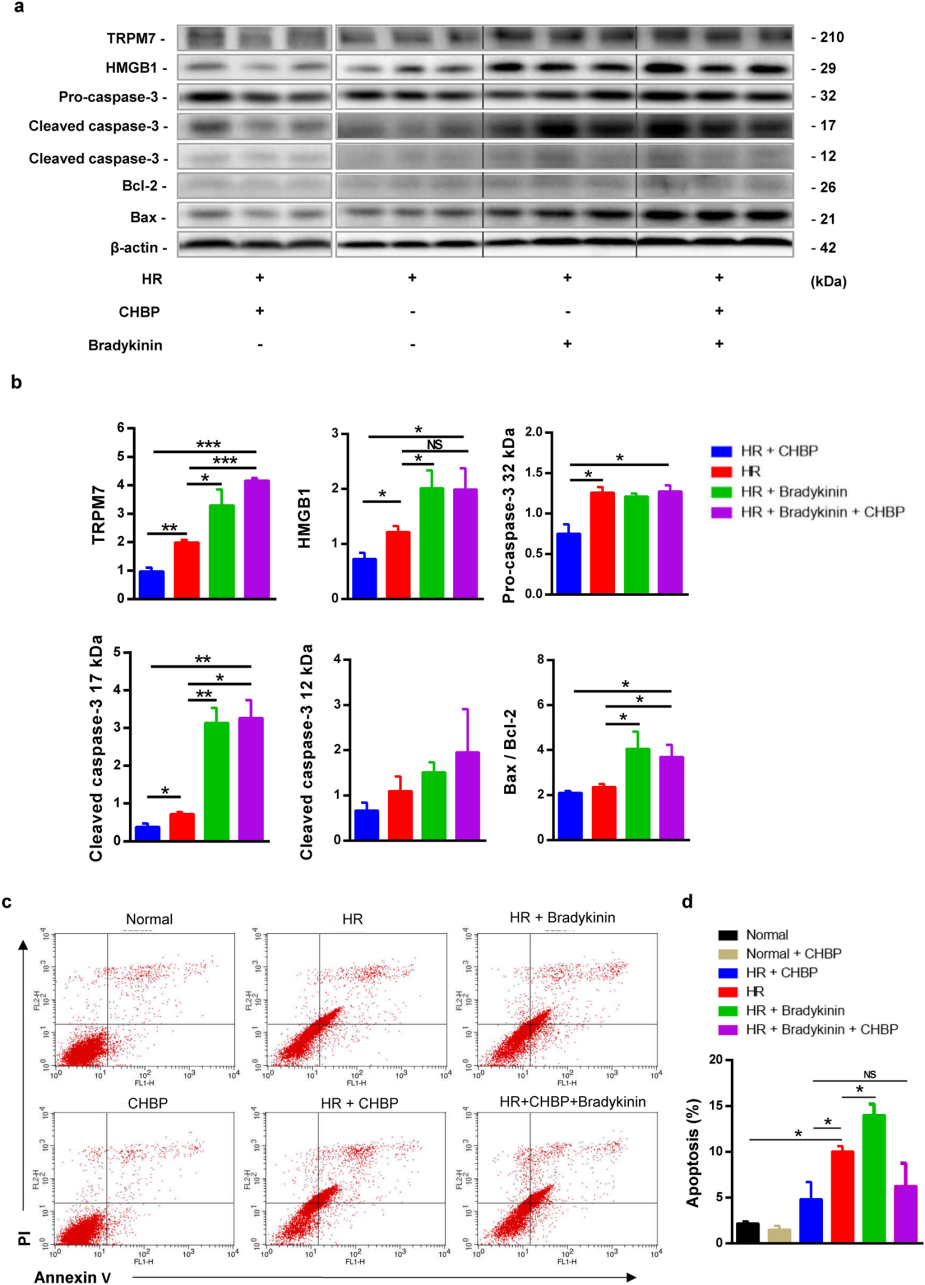
Specific TRMP7 agonist bradykinin aggravated inflammation and apoptosis in TCMK-1 cells. TRPM7, HMGB1, 32, 17 and 12 kDa caspase-3, Bax and Bcl-2 were detected by western blotting (**a**). In HR TCMK-1 cells, CHBP decreased the expression of TRPM7, HMGB1, 32 and17 kDa caspase-3 and Bax/Bcl-2 ratio, while bradykinin and bradykinin + CHBP significantly increased the expression of TRPM7, HMGB1, 17 kDa caspase-3 and Bax/Bcl-2 ratio (**b**). There was no significant difference in 12 kDa caspase-3 between all groups, and in 32 kDa caspase-3 between groups with or without Bradykinin. Apoptotic cells were labelled by Annexin V/PI staining and quantitatively assessed by flow cytometer (**c**). The histograms showed that the increase of early apoptotic cells in the HR group was inhibited by CHBP, but further increased by bradykinin. There was no statistical significance between HR and HR + CHBP + Bradykinin (n = 3, (**d**)).

On the contrary, the effect of specific TRPM7 agonist bradykinin on inflammation and apoptosis was also investigated. The results showed that the activation of TRPM7 increased TRPM7 expression, as well as the inflammation and apoptosis-related protein HMGB1, 17 kDa caspase-3 and Bax/Bcl-2 ratio in HR TCMK-1 cells (Fig. 8a,b).

### TRPM7 contributes to the protective effect of CHBP

To explore the role of TRPM7 on CHBP-induced renoprotection, the joint effect of CHBP and TRPM7 specific agonist bradykinin on inflammation and apoptosis was assessed. First, CHBP reduced TRPM7 expression and then decreased inflammation and apoptosis-related protein HMGB1, 32 and 17 kDa caspase-3 in HR TCMK-1 cells. Further, the activation of TRPM7 partially blocked the effect of CHBP treatment on HMGB1, 17 kDa caspase-3 and Bax/Bcl-2 ratio, but not 32 and 12 kDa caspase-3 (Fig. 8a,b). However, CHBP reduced the percentage of apoptotic cells in HR TCMK-1 cells, which was also not blocked to some extent by TRPM7 activation (Fig. 8c,d).

### TRPM7 correlated with inflammation and apoptosis-related markers

**Figure 9:**
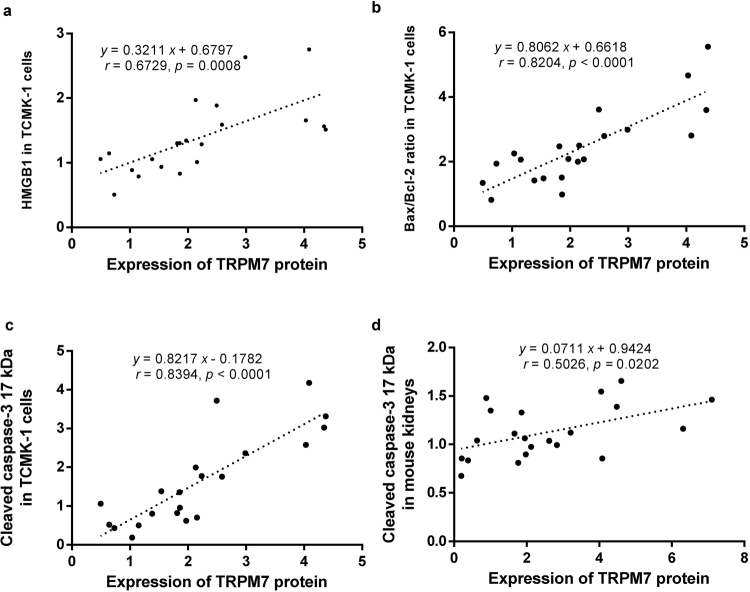
Positive correlations between TRPM7 protein, inflammation and apoptosis-related markers subjected to TRPM7 manipulation. TRPM7 expression was positively related to HMGB1 (**a**) and Bax/Bcl-2 (**b**) in HR TCMK-1 cells, as well as 17 kDa cleaved caspase-3 in both HR TCMK-1 cells (**c**) and mouse IR injury kidneys (**d**).

To further investigate the role of TRPM7 in the renal IR-related injuries and CHBP-induced renoprotection, the correlations between the expression of TRPM7 protein and inflammation and apoptosis-related markers were analyzed after TRPM7 modification by its siRNA and agonist. The results showed that TRPM7 protein expression was positively associated with HMGB1 and Bax/Bcl-2 in TCMK-1 cells, as well as 17 kDa caspase-3 protein both in TCMK-1 cells and mouse kidneys (Fig. 9).

## Discussion

In the present study, the expression of TRPM7 at mRNA and protein level was increased in renal IR-related injuries, but reversed by CHBP. TRPM7 expression was positively associated with injury parameters such as inflammation and apoptosis in HR TCMK-1 cells and mouse IR kidneys, and also tubulointerstital damage and renal function in IR kidneys. Furthermore, silencing TRPM7 by siRNA decreased TRPM7 expression and TRPM7-like current, reduced HMGB1, caspase-3, Bax/Bcl-2 ratio, inflammation and apoptosis in *in vitro* and/or *in vivo* models. In addition, stimulating TRPM7 by bradykinin raised TRPM7 expression, and further increased apoptosis and inflammation in HR TCMK-1 cells. Most interestingly, TRPM7 activation partially blocked CHBP-induced renoprotection upon IR-related injury. Taking together, these data revealed that TRPM7 was involved in not only the renal IR-related injury, but also CHBP-induced renoprotection upon these injures. TRPM7, therefore, could be a potential biomarker for diagnosis and intervention of IR-induced AKI.

TRPM7 is a bifunctional protein characterized by ion channel and kinase activity. In order to investigate the dynamic change of TRPM7, we established a HR model in using both mouse and human renal tubular cell lines, TCMK-1 and HK-2, based on previous studies^37,38^. The up-regulated expression of TRPM7 at mRNA and/or protein level was proved not only in TCMK-1 cells and HK-2 cells exposed to 12-h hypoxia followed by reperfusion for 6 h, 12 h and 24 h; but also in mouse kidneys subjected to 30-min ischemia followed by reperfusion for 12 h, 24 h and 7 d. These findings were consistent with previous studies, in which the expression of TRPM7 was up-regulated in early stage of rat renal IR-related injury^18^.

CHBP, a derivative of erythropoietin, remains tissue protective function without erythropoiesis. Although our previous study revealed that CHBP protected the renal IR-related injury^28,29^, the protective mechanism of CHBP has not been fully evaluated so far. Previous studies showed that EPO pretreatment significantly improved renal dysfunction in a dose-dependent manner, attenuated renal histological damage, reduced TNF-α, IL-1β, IL-6 and ROS production, as well as NF-κB p65 phosphorylation in renal tissues upon IR injury^39^. However, the relation of CHBP and TRPM7 has never been reported. We demonstrated, for the first time, that TRPM7 was involved in cellular HR damage and IR-induced kidney dysfunction, and CHBP reversed up-regulated TRPM7 mRNA and protein in mouse renal IR-related injuries in both *in vivo* and *in vitro* models.

In HR TCMK-1 cells, LDH release is a reliable marker of hypoxic injury correlating well with necrotic cell death^40^. HMGB1, an endogenous ligand for TLR4, is a TLR4-dependent inflammatory mediator in renal IR-related injuries^41^. HMGB1 is identified as one of important DAMP (damage-associated molecular patterns) molecules and released from dead cells^41,42^. HMGB1 is increased in the IR-related injured kidneys in rat native and human transplanted kidneys^43,44^. In order to investigate the damage level and inflammatory responses, we detected the releasing of LDH in the supernatant and HMGB1 in HR TCMK-1 cells. The releasing of LDH and the expression of HMGB1 were increased progressively by prolonged reperfusion time. In addition, there were positive correlations between TRPM7, LDH and HMGB1. These results confirmed the establishment of *in vitro* HR model, as well as TRPM7 involvement in HR TCMK-1 cells.

Renal IR injuries are characterized by functional and structural changes including increased serum creatinine, blood urea nitrogen, cellular inflammation, apoptosis and tubulointerstitial damage^5,28^. Myeloperoxidase (MPO), is a marker of granular cell infiltration in IR kidneys^45,46^. Our previous studies revealed that MPO+ cells, as well as apoptotic cells were increased in IR kidneys^20,28^. This study further showed that TRPM7 expression was increased in IR kidneys from 12 h up to 7 d post reperfusion. In addition, TRPM7 protein was positively associated with serum creatinine, blood urea nitrogen, cellular inflammation, apoptosis respectively, marginally related to tubulointersitial damage scored. The involvement of TRPM7 was further proved in the *in vivo* model of renal IR injury.

Many factors such as oxidative stress, inflammation, necrosis and apoptosis were involved in the pathogenesis of IR-related injuries^47^. One of the core molecular mechanisms is Ca^2+^ overload induced mitochondrial injury, which leads to ROS production, transition pore permeability increase, cytochrome C release and eventual apoptotic cell death^48^. TRPM7, a Ca^2+^ permeable channel, plays a key role in the calcium overload and ROS generation and subsequent inflammation and cell death^12,16,37,49^. It has been reported that TRPM7 contributes to intracellular calcium fluxes that lead to neuronal damage during the long-term oxygen and glucose deficiency^50^, whereas TRPM7 suppression blocked TRPM7 currents, Ca^2+^ uptake, ROS production and anoxic death of neurons^16,51^. Additionally, previous research also demonstrated that the suppression of TRPM7 may alleviate transplant kidney injury^19^. In this study, we first successfully recorded TRPM7-like current in TCMK-1 cells using whole-cell patch-clamping technique. Similar to the previous description, TRPM7-like currents can be identified by their biophysical characteristics, even without applying specific agonists during the electrophysiological recordings^35,36^. Most importantly, we further demonstrated a novel finding that CHBP inhibited the activity of the TRPM7 channel. Therefore we tentatively put forward that TRPM7 might be involved in renal IR-related injuries and CHBP-induced renoprotection through inhibiting calcium overload and ROS production, subsequent reducing inflammation and apoptosis.

Caspase-3 associated with apoptosis and inflammation was involved in renal IR-related injuries. The precursor of caspase-3 could be cleaved into 17 and 12 kDa subunits, both of which contribute to cspase-3 activity^26–29^. In addition, the radio of Bax (apoptotic death accelerator) to Bcl-2 (apoptotic death repressor) determines survival or death following the stimulation of injury mediators^52^. In others and our studies, caspase-3 synthesis and activation, and Bax/Bcl-2 ratio are enhanced by renal IR-related injuries^53^. TRPM7 siRNA silenced TRPM7 expression and reduced TRPM7-like currents, and then decreased HMGB1, caspase-3 synthesis and activation, as well as Bax/Bcl-2 ratio in the *in vitro* and *in vivo* models. In addition, TRPM7 specific agonist bradykinin raised TRPM7 expression and then increased inflammatory responses and apoptosis. Therefore, our data convincingly revealed that TRPM7 was involved in the process of apoptosis and inflammation in renal IR-related injuries. These were consistent with the previous reports that apoptosis was induced by activating caspase-3 and altering Bax/Bcl-2 expression via ROS-dependent pathway^54^. The inhibition of TRPM7 by 2-APB and TRPM7-specific shRNA decreased inflammatory response in asthmatic rats^37^ and TRPM7 was involved in stroke-initiated apoptosis^15,16,51^. On the other hand, recent research demonstrated that the inhibition of TRPM7 induced the cellular apoptosis in prostate cancer^55^. TRPM7 siRNA also increased the rheumatoid arthritis fibroblast-like synoviocytes apoptosis^56^. These controversial results implied that that TRPM7 is involved in apoptosis, which may be through different signal pathways in different cells/models. The precise effect of TRPM7 on apoptosis needs to be carefully assessed in individual settings.

The data from this study also further confirmed that CHBP reduced not only HMGB1, but also the synthesis and activation of caspase-3, as well as Bax/Bcl-2 ratio upon IR-related injuries in TCMK1 cells. At the same time, most importantly, CHBP inhibited TRPM7-like currents, which maintained a regenerative Ca^2+^-dependent ROS production. We, therefore, designed that an experiment using CHBP and TRPM7 agonist jointly to investigate how TRPM7 involved in CHBP-induced renoprotection. It was expectedly found out that activating TRPM7 by bradykinin partially blocked CHBP-induced renoprotection in HR TCMK-1 cells. This provides an additional evidence that the renoprotection of CHBP might be via inhibited TRPM7 in terms of its expression and TRPM7-like current, and then prevent Ca^2+^ overload, ROS production, inflammation and apoptosis.

Inevitably, there were a few limitations in our experimental design. For example, the expression of TRPM7 was detected in TCMK-1 cells exposed to 12-h hypoxia followed by 6, 12, 24-h reoxygenation. However, it was difficult to detect apoptotic cells in the above conditions. Therefore, in order to better confirm the effect of TRPM7 on apoptosis in renal IR-related injuries, the experimental condition was changed to 24-h hypoxia followed by 2-h reoxygenation in TCMK-1 cells for TRPM7 siRNA treatment. Despite these limitations, the present study provided valuable evidence that TRPM7 was involved in renal IR-related injury and CHBP-induced renoprotection.

## Conclusions

For the first time, we demonstrated in details that TRPM7 from its expression and function is involved in not only IR-related acute kidney injury, but also the renoprotection of CHBP. Therefore, our findings shed new lights on the mechanism of renal IR-related injuries and CHBP-mediated renoprotection, and also support TRPM7 being a potential biomarker for diagnostic and therapeutic intervention of renal IR-related injury and beyond.

## Materials and Methods

### Cell culture and HR treatment

A mouse kidney epithelial cell line (TCMK-1, CCL-139) and human renal proximal tubular epithelial cell lines (HK-2, CRL-2190) were purchased from the American Type Culture Collection and maintained in Dulbecco’s modified Eagle’s medium (DMEM)/F12 supplemented with 10% (v/v) fetal bovine serum (FBS, Gibco, Logan, UT, USA), 100 unit/ml penicillin G, 100 µg/ml streptomycin, at 37 °C under humidified atmosphere of 95% air/5% CO_2_^37,38^.

TCMK-1 cells were transfected with 40 nM of small interfering RNA (siRNA) using Lipofectamine^@^ RNAiMAX (Invitrogen, CA, USA) according to the manufacturer’s instructions. Three pairs of double-stranded TRPM7 siRNA, targeting mouse TRPM7 mRNA, were designed and constructed by synthesized in Shanghai GenePharma Co. LTD. These were designated: The sequence of #3239 is 5′-GCAGGACCUUAUGUAAUGATT-3′ (forward) 5′-UCAUUACAUAAGGUCCUGCTT-3′ (reverse). The sequence of #1667 is 5′-GGAGUAAGCAUGCAUAAAUTT-3′ (forward), 5′-AUUUAUGCAUGCUUACUCCTT-3′ (reverse). The sequence of #5326 is 5′-GCUCACAUUUGCCUUUAAUTT-3′ (forward), 5′-AUUAAAGGCAAAUGUGAGCTT-3′ (reverse).

The sequence of #1793 is 5′-GGGUACAAGAUCACUUUAATT-3′ (forward), 5′-UUAAAGUGAUCUUGUACCCTT -3′ (reverse). The negative control siRNA does not target any known mammalian gene and the sequence is 5′-UUCUCCGAACGUGUCACGUTT-3′ (forward), 5′-ACGUGACACGUUCGGAGAATT-3′ (reverse). The transfected cells were cultured at 37 °C for 6 h, and then the following expreriments were performed.

The process of hypoxia (H) reoxygenation (R) was achieved through a hypoxia workstation (H35, DWS, West Yorkshire, UK). Cells were randomly divided into the following groups (at least n = 3 for each time point): (1) control: cells were incubated in normoxic condition (5% CO_2_, 21% O_2_ and 74% N_2_); (2) HR: cells were exposed to hypoxia (5% CO_2_, 1% O_2_ and 94% N_2_) for 12 h, followed by reoxygenation for 6, 12 or 24 h; (3) HR + CHBP: cells pretreated with CHBP were exposed to 12-h hypoxia followed by 6, 12 or 24-h reoxygenation. Thirty nM CHBP were added before exposure to hypoxia. (4) HR + NC siRNA: cells pretreated with NC siRNA were exposed to 24-h hypoxia followed by 2-h reoxygenation (H24R2) (5) HR + TRPM7 siRNA: cells pretreated with TRPM7 siRNA were exposed to H24R2 conditions. (6) HR + Bradykinin: cells pretreated with Bradykinin were exposed to H24R2 conditions. (7) HR + Bradykinin + CHBP: cells pretreated with Bradykinin and CHBP were exposed to the H24R2 conditions. After the respective treatment, cells were washed on ice PBS and harvested to perform further RT-PCR, western blotting and whole cell patch clamp experiments.

### Renal ischemia reperfusion model

This model was generated and its relevant data were previously published^28^. Briefly, male BABL/C mice (8–10 weeks old), weighing 20–22 g, were purchased from Shanghai Slac Lab Animal, Co., Ltd., and bred in an experimental animal room of SPF grade. All animal procedures were performed according to the guidelines of the Care and Use of the Laboratory Animals Ethics Committee of Nantong University and Fudan University, as well as China Association Laboratory Animal Science.

This renal IR model was established as previously published^28^. The mice were anesthetized by intraperitoneal injections of sodium pentobarbital (80 mg/kg). Then, the bitateral renal pedicles were exposed by flank incision and clamped for 30 min. For reperfusion, the clamping was released and the kidney was monitored for color change to confirm blood reflow before suturing the incision. The mice were randomly divided into three groups (n = 6 for each time point): (1) sham group, mice were operated similarly without the renal pedicle clamping. (2) IR group: mice were subjected to ischemia 30 min followed by reperfusion for 12 h, 24 h, 5 d and 7 d. IR injury with PBS intraperitoneally injected. (3) IR + CHBP: IR injury with 8 nmol/kg CHBP intraperitoneally injected at the onset of reperfusion^28^. (4) IR + TRPM7 siRNA: only 48 IR-related injury group treated with 200 μl glucose solution (10%) consisting of 2 mg/kg TRPM7 siRNA and 25 μl Entranster^TM^ -*in vivo* (Engreen Biosystem Co., Ltd, Beijing, China) by tail vein injection 2 h before IR-related injury. (5) IR + NC siRNA: replaced TRPM7 siRNA with 2 mg/kg NC siRNA. The whole blood and kidney samples were required and prepared for further experiment as previously described.

### Quantitative real-time RT-PCR

Total RNA was extracted from the mouse renal tissues or cells with Trizol reagent (Invitrogen, Carlsbad, CA, USA) followed by standard protocols for further processing. Complementary DNA synthesis was performed with 1 µg of total RNA using oligo d (T) 18 Primer and Reverse Transcriptase (Bio-Rad, Hercules, CA, USA). Quantitative RT-PCR (QRT-PCR) analysis was performed in the real time PCR system (CFX96, Bio-Rad) using the commercially available TaqMan® Gene Expression Assay for TRPM7 (Cat: 4331182, Mm00457998_ml) and housekeeping gene β-actin (Cat: 4448484, Mm00607939_ml) with the Fam-labeled and cylophilin Vic-labeled Pre-developed Assay Reagent (Applied Biosystems, Foster City, CA, USA), respectively. We amplified 2 μl cDNA for each sample for each assay in a 20 μl reaction system containing 1x TaqMan Universal PCR Master Mix and 1x gene expression assay with the following cycling conditions: 10 minutes at 95 °C, then 40 cycles of 95 °C for 5 seconds, 60 °C for 30 s and 72 °C for 1 minute. The expression of TRPM7 mRNA in renal tissues or cells normalized with housekeeping gene β-actin was calculated using the 2^−ΔΔCt^ method.

### Western blot analysis

Renal tissues or cells were homogenized in RIPA lysis buffer (Beyotime, Nantong, China) containing PMSF, protease inhibitor and phosphatase inhibitor. Total protein was isolated according to the standard methods^26^. The protein was measured by BCA Protein Quantitation Kit (Pierce, Rockland, USA) and size was separated on sodium dodecyl sulfate-polyacrylamide electrophoresis. Proteins were blotted to polyvinylidene difluoride membranes (PVDF, Roche Diagnostics GmbH, Mannheim, Germany). Blots were blocked with 5% milk and incubated overnight with anti-TRPM7 antibodies (ACC-047, Alomone Labs, Jerusalem, Israel; Ab729, Abcam, Cambridge, UK), monoclonal anti-β-actin (Abcam, 1:10,000) antibodies, and anti-HMGB1 antibody (ab11354, Abcam, 1:1000), anti-caspase-3 antibody (1:1000, Cell Signaling Technology, Beverly, MA), anti-bax antibody (1:1000, Cell Signaling Technology) and anti-bcl-2 antibody (1:1000, Cell Signaling Technology). The horseradish peroxidase-conjugated secondary antibodies (Jackson ImmunoResearch Laboratories, West Grove, PA, USA) were incubated for 2 h at room temperature. Antibody binding was detected by the enhanced chemiluminescent, ECL (Pierce, Rockland, ME, USA) and a Molecular Imager, Chemi Doc, XRS+ System (Bio-Rad, Berkeley, CA, USA). Developed images were semi quantitatively analyzed by scanning volume density using Alpha View Software 3.3 (Cell Biosciences, Inc., Santa Clara, CA, USA). The expression (volume density) of β-actin as the loading control from all samples was averaged. The correlation factor was obtained via the expression of each sample divided by the average. The relative expression of detected protein was obtained by the actual expression divided by the correlation factor to correct the variation of loading.

### Cytotoxicity assay

Cytotoxicity was measured by lactate dehydrogenase (LDH) assay, which was performed as described^40^. Fifty μl medium on 24-well plates growing cells was taken from each well, the cell supernatants was detect by automatic biochemistry analyzer (Siemens, Berlin, Germany).

### Annexin V/PI assay

Cell apoptosis was analyzed by Annexin V-FITC Apoptosis Detection Kit (BD Pharmingen, San Diego, CA, USA). TCMK-1 cells were collected by centrifugation, washed in PBS and then resuspended in 200 μl 1x binding buffer containing 10 μl of Annexin V-FITC and incubated for 15 min at room temperature in the dark. 5 μl of PI and 200 μl of binding buffer were added to each example and also incubated for 5 min at the same condition. The cells were analyzed by BD FACS Calibur flow cytometer (BD Biosciences). In each example, a minimum of 10,000 cells were counted, data was performed using Cell Quest software (BD Biosciences).

The results were shown as quadrant dot plots with intact cells (Annexin V−/PI −), early apoptotic cells (Annexin V+/PI−), late apoptotic cells or necrotic cells (Annexin V+/PI+). The number of each kind of cells was expressed as percentages of the number of total stained cells.

### TRPM7-like current recordings in TCMK-1 cells

The whole-cell configuration of the patch–clamp technique was used in the study according to our previous methods^36^. Patch-clamp experiments were performed in the whole-cell configuration at 21-25 °C using TCMK-1 cells grown on glass coverslips and maintained in an Mg^2+^-free extracellular solution of the following composition (in mM): 140 NaCl, 5.4 KCl, 33 Glucose, 25 HEPES and 1.3 CaCl2, with pH adjusted to 7.4 using NaOH. Patch electrodes were fabricated using micropipette puller (P-97, Sutter Instrument Co., Novato, CA, USA). The resistance was 3-5 MΩ when filled with Mg^2+^-free internal solution of the following composition (mM): 140 CsCl, 10 HEPES, 2 TEACL, 5 EGTA, and 1 CaCl2, with pH adjusted to 7.4 using CsOH. High-resolution current recordings were acquired using a patch-clamp amplifier system (EPC 10, Heka, Germany). Following whole-cell formation, 2-s voltage ramps between −80 mV and +100 mV were delivered from a holding potential of −60 mV. Currents were filtered at 2 kHz and digitized at 10 kH using EPC-10 acquisition system (Heka, Germany). Capacitive currents and series resistance were determined and corrected prior to each voltage ramp, using the automatic capacitance compensation of EPC 10. Voltage-gated Ca^2+^ channels were blocked using 5 mM nimodipine. For each TCMK-1 cell, current values at different time points (at +100 mV) were measured immediately.

### Statistical analyses

All data are representative of at least three independent experiments, the results are expressed as mean ± SD. Statistical analysis using SPSS 18.0 software (SPSS Inc., Armonk, NY, USA) was performed with the two-tailed independent Student’s *t*-test for two groups after the demonstration of homogeneity of variance with the F test or one-way ANOVA for more than two groups. The Scheffe test was used for *post-hoc* analysis. P < 0.05 was considered statistically significant.
